# Vitamin D concentration and focal brain atrophy in older adults: a voxel‐based morphometric study

**DOI:** 10.1002/acn3.50997

**Published:** 2020-03-09

**Authors:** Pauline Ali, Matthieu Labriffe, Nastassia Navasiolava, Marc‐Antoine Custaud, Mickaël Dinomais, Cédric Annweiler, the SAM group

**Affiliations:** ^1^ Laboratoire Angevin de Recherche en Ingénierie des Systèmes EA7315 University of Angers – Université Angers Angers France; ^2^ Department of Physical and Rehabilitation Medicine Angers University Hospital Angers France; ^3^ Department of Radiology Angers University Hospital Angers France; ^4^ Clinical Research Center Angers University Hospital Angers France; ^5^ MITOVASC Institute UMR CNRS 6015 UMR INSERM 1083 University of Angers Angers France; ^6^ Department of Geriatric Medicine Angers University Hospital Angers France; ^7^ Memory Clinic Angers University Hospital Angers France; ^8^ Research Center on Autonomy and Longevity Angers University Hospital Angers France; ^9^ UPRES EA 4638 University of Angers Angers France; ^10^ Department of Medical Biophysics Schulich School of Medicine and Dentistry Robarts Research Institute University of Western Ontario London Ontario Canada

## Abstract

Vitamin D is involved in brain health and function. Our objective was to determine whether lower 25‐hydroxyvitamin D (25OHD) concentration was associated with focal brain volume reduction in older adults. Serum 25OHD concentration was measured among 53 older adults (72 ± 5 years; 38% female; mean 25OHD = 67.3 ± 20.8 nmol/L). Gray matter volume (GMV) was automatically segmented using voxel‐based morphometry with CAT12 software. Covariables were age, gender, education, total intracranial volume, and season. Serum 25OHD was positively associated with GMV in left calcarine sulcus (*P* < 0.05, TFCE, FWE‐corrected). We found atrophy of the calcarine sulcus with lower 25OHD concentrations in older adults.

## Introduction

Besides its classical function of bone metabolism regulation, vitamin D exhibits multiple biological targets mediated by its nuclear hormone receptor, the Vitamin D Receptor (VDR). VDRs are present in neurons and influence a number of physiological processes in the nervous system including the synthesis of neurotrophic agents, anti‐inflammatory factors, and antioxidants.^1^, ^2^ Consistently, lower serum concentrations of 25‐hydroxyvitamin D (25OHD) have been associated in older adults with brain volumetric changes, including lower whole‐brain volume and larger lateral ventricles,^3^ with possible consequences on cognitive decline^4^ and fall risk.^5^ However, it remains unelucidated thus far which brain regions undergo atrophy in the case of hypovitaminosis D, and would explain the decrease in the overall volume of the brain in this population. Better understanding the neuroanatomical correlates of vitamin D status would yet offer a powerful mechanism to act on brain changes in older adults and maintain function later in life. We had the opportunity to examine the associations of serum 25OHD concentration with the volume of gray matter (GM) in a cohort of community‐dwelling older adults, the IRMarche study.^6^ The objective of this cross‐sectional analysis was to determine whether lower 25OHD concentrations were associated with focal brain volume reductions.

## Methods

### Participants

We studied participants from the IRMarche study enrolled between 2015 and 2017. Main inclusion criteria were: age over 60 years, absence of clinical dementia according to the consensual DSM IV criteria, and no neurological, depression, or orthopaedic disorders. We excluded participants with contraindication to MRI. All participants included in the present analysis received a full medical examination, a neuropsychological assessment, a blood test with 25OHD assay, and a 3.0‐Tesla magnetic resonance imaging (MRI) scan of the brain.

### Serum 25OHD concentration

Blood samples were collected from resting participants at the same time as the brain imaging acquisition. Serum 25OHD concentration, which reflects the vitamin D status, was measured using radioimmunoassay (DiaSorin Inc., Stillwater, MN) in nmol/L at the University Hospital of Angers, France. Vitamin D deficiency was defined for 25OHD concentrations ≤50 nmol/L according to the definition of the Institute of Medicine (to convert to ng/mL, divide by 2.496).^7^

### MRI procedure

#### MRI data acquisition

MRI data were acquired on the same 3.0‐Tesla MRI scanner (MAGNETOM Trio Tim system, Siemens, Erlangen, Germany) at the University Hospital of Angers, France, using a standard protocol. Acquisition parameters for sequences including a high‐resolution 3D T1‐weighted volume using a magnetization‐prepared rapid acquisition gradient‐echo sequence, covering the whole brain (cerebellum included) were: 176 slices, repetition time (TR) = 2300 msec, echo time (TE) = 4.18 msec, slice thickness = 1 mm, field of view (FOV) = 256 × 256, flip angle = 9, voxel size: 1 × 1 × 1 mm^3^.

#### Voxel‐based morphometry

Voxel‐based morphometry across the entire brain were conducted with the Computational Anatomy Toolbox 12 (CAT12) (http://www.neuro.unijena.de/cat) implemented in the Statistical Parametric Mapping 12 (SPM12) software (https://www.fil.ion.ucl.ac.uk/spm/software/spm12/) using a 2017a Matlab platform (The MathWorks, Natick, MA, USA), following the standard procedure. First 3D T1 volumes were segmented in native‐space to obtain three tissues classes: GM, white matter, (WM) and cerebrospinal fluid (CSF).^8^ Then, nonlinear modulation GM and WM volumes were aligned using the default DARTEL (diffeomorphic anatomical registration using exponential Lie algebra) template and were spatially normalized into the Montreal Neurological Institute space (ICBM).^9^ Finally, images were smoothed by an isotropic Gaussian kernel of 8mm full‐width at half‐maximum (FWHM). All data were inspected and visually checked after the automated procedure by two authors (PA and MD).

### Covariates

Age, gender, high education level, season, and total intracranial volume (TIV) were included as potential confounders for the analysis. Education level was reported with a structured questionnaire. Participants who passed at least the Elementary School Recognition Certificate were considered to have higher education level compared with those who did not. The TIV was approximated for each participant by calculating the sum of GM, WM, and CSF maps obtained from the preprocessing steps.

### Statistics

Statistical analyses were performed using Tanagra 2.0 software (http://eric.univ-lyon2.fr/~ricco/tanagra/fr/tanagra.html), Excel (Microsoft office Professional Plus 2013) and CAT12. For neuroimaging, smoothed, modulated and normalized data were used. With CAT12, morphometric data from participants were associated with a linear regression model with 25OHD concentration. We used threshold‐free cluster enhancement (TFCE) for a combined nonparametric analysis about height and size of the effects (http://www.neuro.uni-jena.de/tfce), and applied FWE (family wise error) corrected threshold of *P* < 0.05 to control for multiple comparisons. Anatomy toolbox 2.1 was used for anatomical localizations.^10^

### Ethics

The study was conducted in accordance with the ethical standards set forth in the Helsinki Declaration (1983). The entire study protocol was approved by the Angers Ethical Review Committee (Comité de protection des personnes, CPP ouest II, Angers, France, n° A.C D 2014‐A01593‐44, n° CPP: 2014/32). Written informed consent was obtained at enrollment.

## Results

Fifty‐three participants (mean ± SD, 72 ± 5 years; 38% female) met the selection criteria and were included in the present analysis. The mean serum 25OHD concentration was 67.3 ± 20.8 nmol/L [95% confidence interval (CI): 62–73] (range, 28–113 nmol/L) among studied participants, and the mean TIV was 1533 ± 154 cm^3^ [95% CI: 1492–1574] (range, 1212–1822 cm^3^). Twenty‐nine participants (54%) had a high level of education. Twenty subjects (38%) were assessed (blood and MRI) in the autumn, 16 (30%) in the winter, 12 (23%) in the spring, and 5 (9%) in the summer.

**Figure 1:**
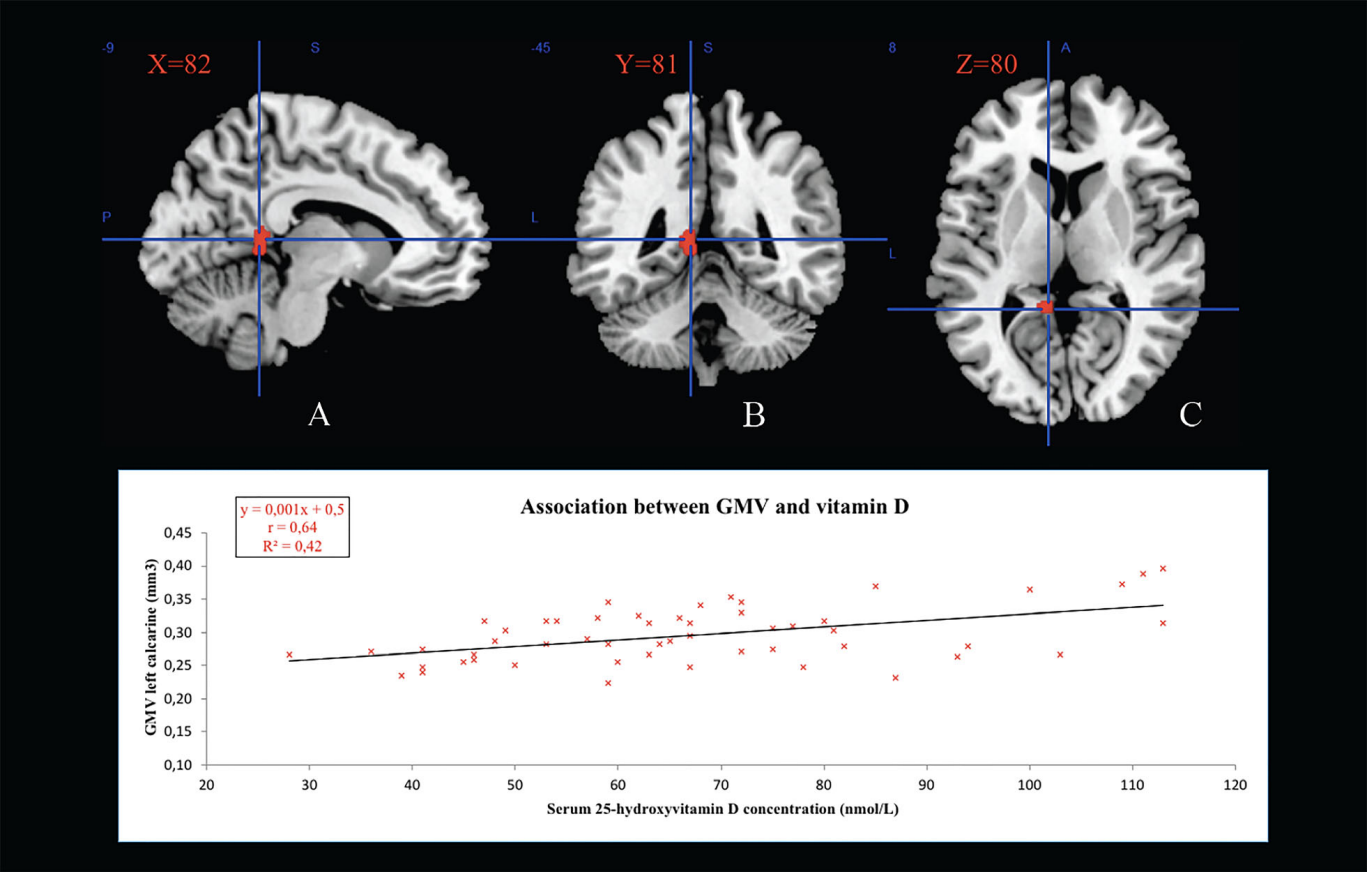
Gray matter region showing a positive association with serum 25‐hydroxyvitamin D concentration (red color). GMV: gray matter volume. The statistical map was co‐registered and superimposed on 3D‐T1 (A) sagittal slice (MNI coordinates: X = 82), (B) coronal slice (MNI coordinates: Y = 81), and (C) axial slice (MNI coordinates: Z = 80) (MNI T1 template available on MRICRON software; TFCE from 10 to 1397). Results are showed with a significance of *P* < 0.05 corrected for multiple comparisons (TFCE, FWE‐corrected).

Figure 1 illustrates the brain area that were positively associated with the serum 25OHD concentration after adjusting for potential confounders. The VBM analysis identified one cluster of 110 voxels located in the left calcarine sulcus (MNI coordinates (xyz) [−11 −44 8], TFCE = 1366, *P* < 0.05 FWE‐corrected). No other regions were positively or negatively associated with serum 25OHD concentration.

## Discussion

Our results show that, among community‐dwelling older adults, lower serum 25OHD concentration was associated with lower GMV in the left calcarine sulcus specifically. This finding provides insights into elucidating the role of vitamin D in older adults’ brain, notably in the treatment of visual stimuli.

A growing body of preclinical evidence supports a role for vitamin D in brain health and morphology. In particular, vitamin D appears to have a trophic function in the differentiation and maturation of neurons by controlling the rate of mitosis and the levels of neurotrophins such as the Nerve Growth Factor (NGF) or the neurotrophin 3.^1^ Vitamin D also promotes neuronal calcium homeostasis by downregulating the expression and density of calcium channels, and via the synthesis of calcium‐related cytoplasmic proteins such as parvalbumine or calbinding protein.^1^ As an illustration, in one study, vitamin D treatment protected neurons against amyloid‐β peptide toxicity by downregulating calcium channels and inducing NGF release.^11^ Vitamin D also has antioxidant effects in cultures of rat mesencephalic cells.^12^ Finally, vitamin D exhibits anti‐inflammatory effects in the brain,^13^ consistent with reduced inflammatory brain injury following vitamin D repletion.^14^

The identification of focal brain atrophy related to vitamin D has received little attention to date. Previous studies mostly focused on the changes of whole‐brain volume,^15^ ventricular volume,^16^ and hippocampal volume^17^, ^18^ according to the vitamin D status.

The present analysis provides additional information on the morphological brain changes related to vitamin D, by providing that some specific subvolumes of GM are affected with lower (i.e., worse) 25OHD concentration. Another recent study evokes that vitamin D deficiency is associated with thinner cingulate cortex.^19^

We found that lower 25OHD concentrations were associated with GM atrophy in the left calcarine sulcus. Since the calcarine sulcus is part of the primary visual cortex, which is essential for the conscious treatment of visual stimuli, our result is in agreement with previous epidemiological findings that hypovitaminosis D is associated with reduced visual performance among older adults.^20^ This association was tentatively explained by the onset of age‐related macular degeneration (AMD),^21^ glaucoma,^22^ or optic neuropathy^23^ in the case of hypovitaminosis D. However, no causal relationship has been established thus far, and the result of the present study suggests a novel mechanism possibly explaining how vitamin D influences vision, by an effect on the primary visual cortex trophicity rather than on the eye.

Some potential limitations of our study should be considered. Firstly, the study cohort was restricted to community‐dwelling older adults recruited in one single research center who might be unrepresentative of all older adults. Secondly, although we were able to control for important characteristics that could modify the association, residual potential confounders such as the eye condition or reduced sunlight exposure might still be present. Thirdly, the cross‐sectional design did not allow any causal inferences making it difficult to determine whether the focal brain atrophy precipitated hypovitaminosis D or whether hypovitaminosis D played a role in the onset of focal brain atrophy. Fourthly, VBM has been criticized because of segmentation and normalization defects. Segmentation of brain into GM and WM is a major difficulty due to partial volume effects at the boundary between GM and WM, and because of mislabeling. We minimized these limitations by using SPM12 software that performs better than previous versions.

In conclusion, we found that lower serum 25OHD concentration was associated in older adults with GM atrophy in the calcarine sulcus, part of the primary visual cortex. This finding highlights for the first time that specific brain areas are altered with hypovitaminosis D, and helps understanding the effects of vitamin D on brain health and function in older adults, notably the brain treatment of visual stimuli. Further prospective observational cohorts and randomized clinical trials, preferentially on a variety of adult populations, are needed to clarify whether those with hypovitaminosis D are more likely to experience loss of GM in the visual cortex, and whether vitamin D supplementation could prevent or limit this change.

## Author Contributions

Each author has made substantial contributions to the conception or design of the work; or the acquisition, analysis, or interpretation of data; or the creation of new software used in the work; or have drafted the work or substantively revised it; AND has approved the submitted version; AND agrees to be personally accountable for the author’s own contributions and for ensuring that questions related to the accuracy or integrity of any part of the work, even ones in which the author was not personally involved, are appropriately investigated, resolved, and documented in the literature. Mickaël Dinomais has full access to all of the data in the study, takes responsibility for the data, the analyses and interpretation, and the conduct of the research, and has the right to publish any and all data, separate and apart from the attitudes of the sponsor. Study concept and design: Mickaël Dinomais, Cédric Annweiler, Marc‐Antoine Custaud. Acquisition of data: Matthieu Labriffe, Mickaël Dinomais, Nastassia Navasiolava. Analysis and interpretation of data: Pauline Ali, Mickaël Dinomais, Cédric Annweiler. Drafting of the manuscript: Pauline Ali, Mickael Dinomais, Cédric Annweiler. Critical revision of the manuscript for important intellectual content: Matthieu Labriffe, Mickael Dinomais, Nastassia Navasiolava, Pauline Ali, Marc‐Antoine Custaud. Obtained funding: Mickaël Dinomais. Statistical expertise: Pauline Ali, Mickaël Dinomais. Administrative, technical, or material support: Nastassia Navasiolava, Mickaël Dinomais, Marc‐Antoine Custaud. Study supervision: Mickaël Dinomais.

## Conflict of Interest

C. Annweiler serves as an editor for Maturitas. All authors declare they do not have any other financial and personal conflicts of interest with this manuscript.
